# Monte Carlo simulation of the effect of melanin concentration on light-tissue interactions in transmittance and reflectance finger photoplethysmography

**DOI:** 10.1038/s41598-024-58435-7

**Published:** 2024-04-08

**Authors:** Raghda Al-Halawani, Meha Qassem, Panicos A. Kyriacou

**Affiliations:** Research Centre for Biomedical Engineering, City, University of London, London, UK

**Keywords:** Biomedical engineering, Biophotonics, Quality of life

## Abstract

Photoplethysmography (PPG) uses light to detect volumetric changes in blood, and is integrated into many healthcare devices to monitor various physiological measurements. However, an unresolved limitation of PPG is the effect of skin pigmentation on the signal and its impact on PPG based applications such as pulse oximetry. Hence, an in-silico model of the human finger was developed using the Monte Carlo (MC) technique to simulate light interactions with different melanin concentrations in a human finger, as it is the primary determinant of skin pigmentation. The AC/DC ratio in reflectance PPG mode was evaluated at source-detector separations of 1 mm and 3 mm as the convergence rate (Q), a parameter that quantifies the accuracy of the simulation, exceeded a threshold of 0.001. At a source-detector separation of 3 mm, the AC/DC ratio of light skin was 0.472 times more than moderate skin and 6.39 than dark skin at 660 nm, and 0.114 and 0.141 respectively at 940 nm. These findings are significant for the development of PPG-based sensors given the ongoing concerns regarding the impact of skin pigmentation on healthcare devices.

## Introduction

Photoplethysmography (PPG) is a non-invasive optical technique widely used in the study and monitoring of the pulsations associated with changes in blood volume in a peripheral vascular bed. Over the last thirty years, there has been a significant increase in the number of published articles on PPG, describing both basic and applied research^1^. Throughout these publications the PPG has been hailed as a non-invasive, low cost, and simple optical measurement technique applied at the surface of the skin to measure physiological parameters. The popularity of this topic can be attributed to the realisation that PPG has important implications for a wide range of applications, including amongst many, blood oxygen detection, cardiovascular system assessment, and vital sign monitoring. In addition, the significant contribution of PPG in wearable devices has exponentially elevated the popularity and usability of PPG.

There is currently a large body of literature contributing new knowledge on the relation of the PPG pulse morphology, pulse wave analysis and pulse features extraction with the physiological status of peripheral blood vessels, such as aging, stiffness, blood pressure and compliance, microvascular disease, amongst others^2–5^. There are also significant efforts in the utilisation of the PPG for the detection of heart arrhythmias such as atrial fibrillation (AF). Researchers are continuing to strive in combining the PPG sensory capabilities of wearables, such as smartwatches, with artificial intelligence (AI) in delivering ubiquitous health monitoring solutions that go beyond the current available heart rate wearables.

Despite the wide spread utilisation of PPG, PPG based technologies such as pulse oximetry and various wearable devices have limitations. These limitations can be impacted by physiological factors such as low peripheral perfusion, or technical factors such as motion artifact. For example, over the years we have witnessed many improvements in the design of pulse oximeters in an effort to mitigate some of the well know limitations of PPG. However, some challenges still remain and efforts to address them continue.

Another unresolved limitation of PPG is the effect of skin pigmentation on the signal and its impact on PPG based applications such as pulse oximetry. The causes of inaccuracy imposed by skin pigmentation on PPG is still unclear and requires further research to, for instance, understand the biases seen in finger and cerebral oximetry in individuals with darker skin pigmentation^6,7^. Some emerging approaches include PPG-imaging, machine learning and artificial intelligence to obtain more comprehensive information about skin pigmentation, and potentially improve the accuracy of skin pigmentation estimation within and between individuals^8,9^. However, to understand the possible causes of differential outputs in PPG measurements amongst mixed populations using an in silico method such as Monte Carlo simulations, is a faster preliminary approach. Therefore, the motivation of this study is to explore the light-tissue interactions through a simulated anatomy that accounts firstly for the differences in skin pigmentation before including other confounding factors for application-based studies.

The interchangeable relationship between experimental and in-silico studies allows us to predict and cross validate the outcomes of a biological system. Monte Carlo models have been extensively used as a computational tool to gain a comprehensive insight into light-tissue interactions. It is a mathematical technique which involves modelling the probability of different outcomes with random variables. With its ability to replicate the stochastic nature of turbid media such as biological tissue, it is used to visualise, explore, and analyse the optical interactions of multiple tissue layers within a specific region of interest (ROI).

Meglinski and Doronin^10^ state the advantages of the MC method over other methods used to model light-tissue interactions. For instance, in comparison to the random walk model, it considers biological heterogeneity, which arises from the non-uniform composition of blood and skin pigment distributions, and more. Other advantages include the ability to produce accurate results whilst offering flexibility with regards to the size, shape and position of the optical source and detector. Moreover, the diffusion theory neglects the ‘ballistic’ nature of photon propagation in a moderate dominated by scattering^11^, which is an essential representation of biological tissue. Nevertheless, the main drawback with MC simulations is its long computation time, however this is not considered to be a fundamental problem owing to advances in computer technology.

The behaviour of light in human skin using the Monte Carlo technique has been explored in the literature^12–25^, with a main focus on the effect of melanin and skin pigmentation on PPG^20–25^. Computational studies are vital for understanding the effect of optical (e.g. wavelength, beam size, etc.) and non-optical (e.g. thickness of the tissue layer) input parameters to achieve optimal/desired bio-optical outcomes. However, more relevant to the scope of this study, the latter studies have found that higher levels of melanin concentration result in relatively greater magnitude of light absorption than different concentrations of blood. The effect of melanin concentration on the PPG signal quality has been evaluated by Ajmal et al.^20^ and Boonya et al.^25^, who found a 6.6 ± 0.5% and 4.66% decrease in the pulsatile-non-pulsatile ratio (AC/DC) in non-obese individuals between light and dark skin, respectively. As they focused on measuring different parameters such as blood pressure and heart rate derived from PPG, different wavelengths were utilised in their models. Hence, following previous work on the effect of melanin concentration in a monolayer MC model of the skin^22^, this study aims to provide a more realistic representation of light-tissue interactions in a simulated human finger at different melanin concentrations, wavelengths, and PPG sensor configurations to understand its effect on the PPG signal.

## Materials and methods

A finger with a total thickness (*t*) of 1.3 cm was adapted from Chatterjee & Kyriacou^12^, comprising of skin (*t* = 0.95 mm), fat (*t* = 0.5 mm), and muscle with a cylindrical bone (*t* = 10.1 mm). The same volume fractions of water and blood, and the protocol for achieving pulsatility in all the dermal sublayers were implemented^26^ at a constant oxygen saturation level of 100%. The model was simulated for the combinations of the selected wavelengths and cardiac phases; red light (660 nm) during systole and diastole, infrared light (940 nm) during systole and diastole. To simulate systole, blood volume was assumed to be two times more than diastole in the dermal layers to mimic blood volumetric changes during cardiac pulsations. The volume fractions of melanosomes (${v}_{mel}$) was adjusted to mimic three skin type groups from the Fitzpatrick scale excluding skin type III, corresponding to light, moderate, and dark skin^27^ (Table 1). Skin phototype III was not simulated as the melanosome volume for this scale was not available, which was acceptable for the purposes of this study as the analysis of the light-tissue interactions between distinct skin groups could still be achieved. Using this information, the absorption coefficient of the epidermal layer (${\mu }_{a\_epi}$) was calculated using the following equation^28^:

**Table 1:** Volume fraction of melanosomes in the human epidermis for three different pigmentation groups^27^.

Skin type	Estimated Fitzpatrick skin type	Melanosome volume (${v}_{mel}$, %)
Light	I-II	2.55
Moderate	IV-V	15.5
Dark	VI	30.5

Light skin is assumed to represent Caucasian populations, moderate skin for Asian and Latin populations, and dark skin for African/Black populations.

1 $${\mu }_{a\_epi} \left[{{\text{mm}}}^{-1}\right]=\left({v}_{mel}\right)\left(6.6\times {10}^{10}\right)\left({\uplambda }^{-3.33}\right)+\left({v}_{W}\right)\left({\mu }_{aW}\right)\left(1-{v}_{mel}-{v}_{W}\right)\left(0.244+85.3{{\text{e}}}^{\left(-\frac{\uplambda -154}{66.2}\right)}\right).$$

**Figure 1:**
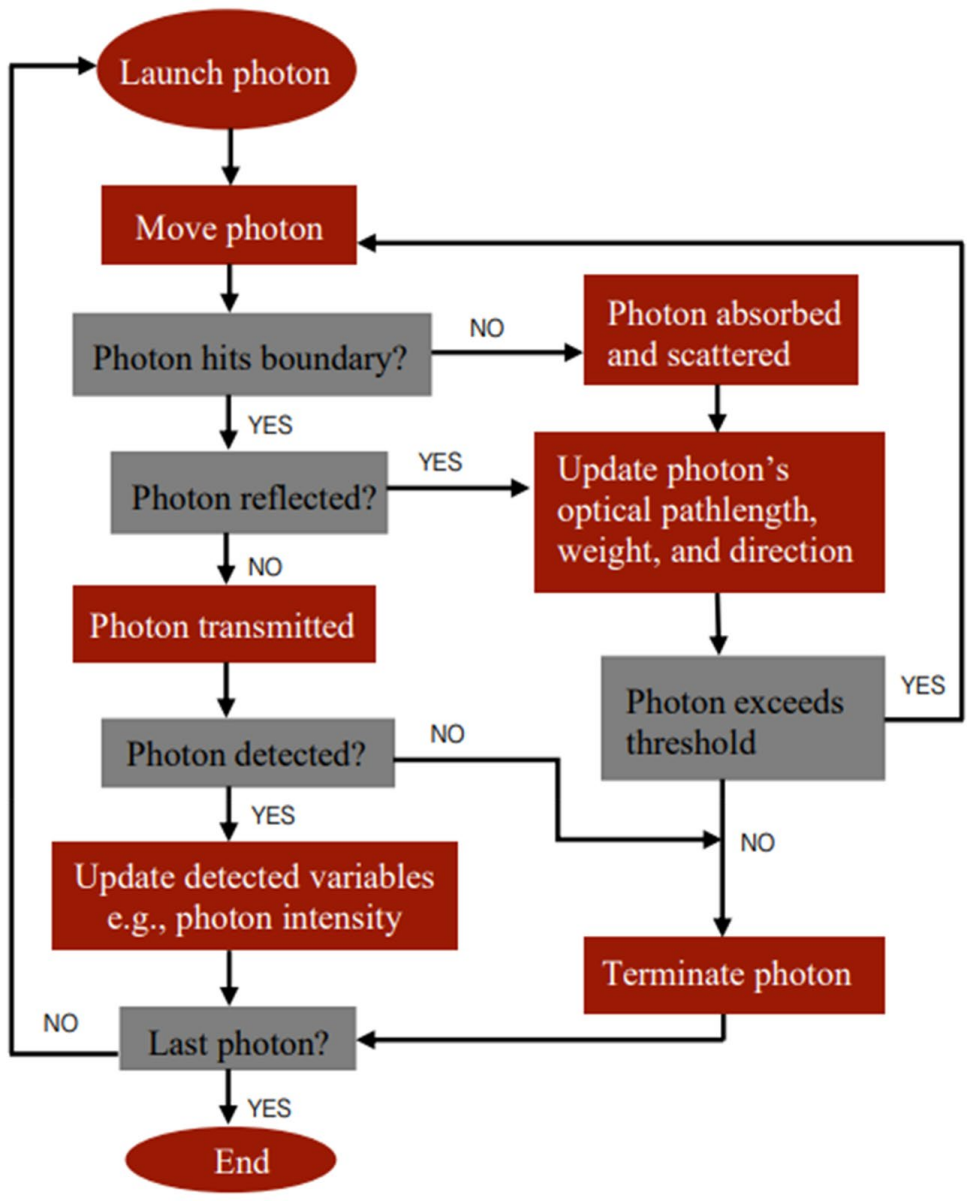
Monte Carlo algorithm adapted from^12,29,30^, which governs the movement of photons between the source and detector in both transmittance and reflectance PPG modes.

**Figure 2:**
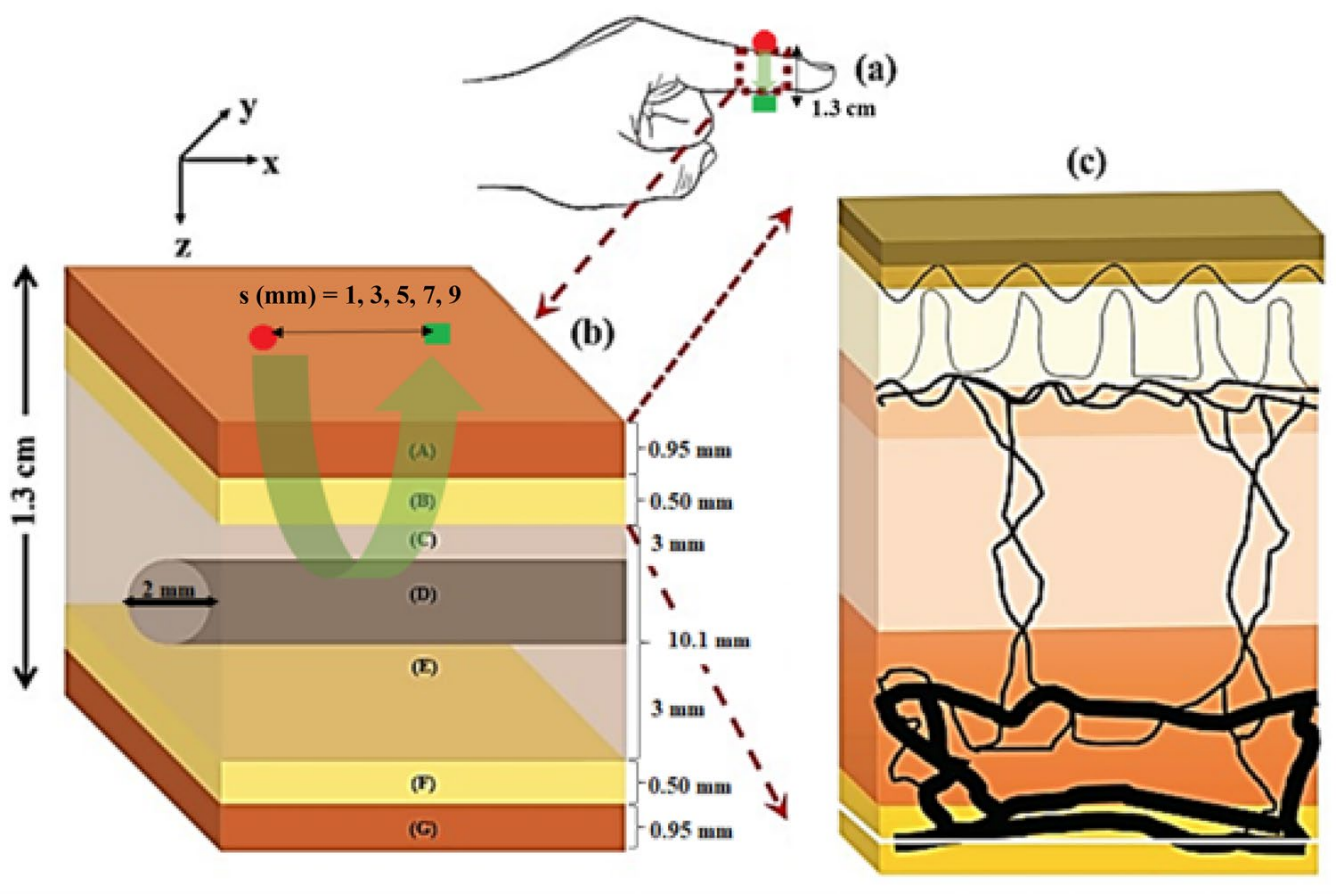
Block representation of the finger. (**a**) The region of interest with transmittance sensor configuration (source and detector placed opposite to the each other across the finger). (**b**) Illustrates the different layers of the finger with their corresponding thicknesses: A & G are skin layers, B & F are fat layers, and C & E are muscle layers containing a cylindrical bone D. The reflectance sensor configuration is also illustrated (source and detector are placed adjacent to each other) with source-detector separations between 1 and 9 mm in 2 mm increments. (**c**) Skin structure comprising of 6 layers – stratum corneum, epidermis, papillary dermis, upper blood net dermis, reticular dermis, deep blood net dermis, connected to the sub dermis (fat layer). Green arrows show the overall trajectory of the photons in both PPG modes.

**Figure 3:**
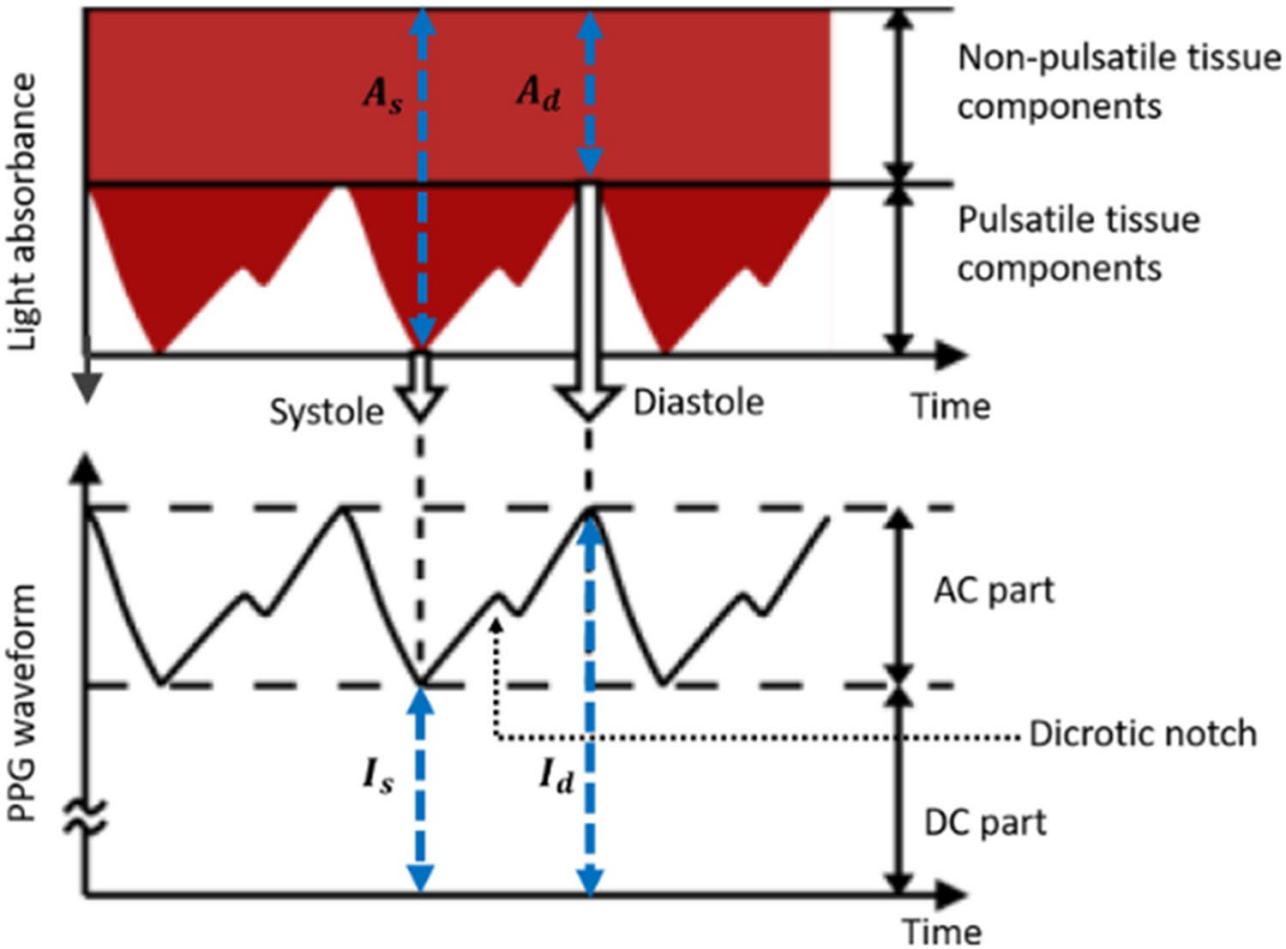
Schematic of the PPG waveform produced as a result of light absorbance during systole and diastole (A_s_ and A_d_). The corresponding light intensities are denoted by I_s_ and I_d_, used for the calculation of the pulsatile AC component and non-pulsatile DC component from the Monte Carlo simulation.

The principles of the MC method for modelling light transport in multi-layered tissues, including all the mathematical equations used to create the current model, are described by Jacques et al.^29^ and Wang et al.^30^. These steps were adapted to create a MC algorithm (Fig. 1) implemented in MATLAB, providing a set of conditions to govern the movement of photons at 660 nm and 940 nm through the finger model as they undergo absorption, scattering, reflection, and transmission. Hence, all the optical properties of the layers and constituents of a human finger were acquired from the literature at these two wavelengths^31–37^ (Table 2). The position of the photons were represented by Cartesian coordinates ‘*x*’, ‘*y*’, and ‘*z*’, and the polar coordinate system defined their direction cosines ‘${u}_{x}$’, ‘${u}_{y}$’, and ‘${u}_{z}$ ’to account for anisotropic scattering in the corresponding three axes relative to a vector ‘$\overrightarrow{{\text{r}}}$’^12,22^. To simulate transmittance and reflectance modes of PPG, the photodetector was placed opposite and adjacent to the light source with a Gaussian beam distribution, respectively. In reflectance mode, the source-detector separation (*s*) was varied between 1 and 9 mm in increments of 2 mm by changing the distance of the photodetector relative to the light source in the ‘*x*’ direction, i.e. across the width of the finger model. The multi-layered model of the finger and the source-detector configurations are illustrated in Fig. 2. Lastly, to calculate the magnitude of the AC and DC PPG output at a single point in time, the generated intensities from the MC simulation were used to calculate transmittance and reflectance, as presented in Fig. 3. The AC component was calculated by subtracting the systolic intensity by the diastolic intensity, and the DC component was given by the systolic intensity:

**Table 2:** Optical properties of all the tissue components of a human finger at 660 nm and 940 nm, as obtained from the literature^31–37^.

Tissue component	${\upmu }_{{\text{a}}} ({{\text{mm}}}^{-1}$)		${\upmu }_{{\text{s}}} ({{\text{mm}}}^{-1}$)		g	
	660 nm	940 nm	660 nm	940 nm	660 nm	940 nm
Skin	Optical properties vary with cardiac phase		25.62	15.68	0.91	0.94
Fat	0.0104	0.0170	6.20	5.42	0.90	0.90
Muscle	0.0816	0.0401	8.61	5.81	0.88	0.91
Bone	0.0351	0.0457	34.45	24.70	0.92	0.93
Oxyhaemoglobin	0.15	0.65	–	–	–	
Deoxyhaemoglobin	1.64	0.43	–	–	–	
Water	0.00036	0.02674	–	–	–	
Melanin	26.944	8.299	–	–	–

2 $$AC= {I}_{d}-{I}_{s},$$

3 $$DC= {I}_{s},$$

## Results

This section presents the results for the analysis of the simulated light-tissue interactions in light, moderate, and dark skin, in transmittance PPG (Sect. “Transmittance PPG”) and reflectance PPG (Sect. “Reflectance PPG”). The calculated absorption coefficients of the epidermis for all skin types are presented in Table 3, calculated using Eq. (1). These results correlated with the data produced by Patwardhan et al.^38^.

**Table 3:** Absorption coefficients (${\upmu }_{{\text{a}}}$) of light, moderate, and dark epidermis at 660 nm and 940 nm.

	Light skin	Moderate skin	Dark skin
Red light (660 nm)	0.727 ${{\text{mm}}}^{-1}$	4.21 ${{\text{mm}}}^{-1}$	8.24 ${{\text{mm}}}^{-1}$
Infrared light (940 nm)	0.229 ${{\text{mm}}}^{-1}$	1.30 ${{\text{mm}}}^{-1}$	2.54 ${{\text{mm}}}^{-1}$

### Transmittance PPG

**Figure 4:**
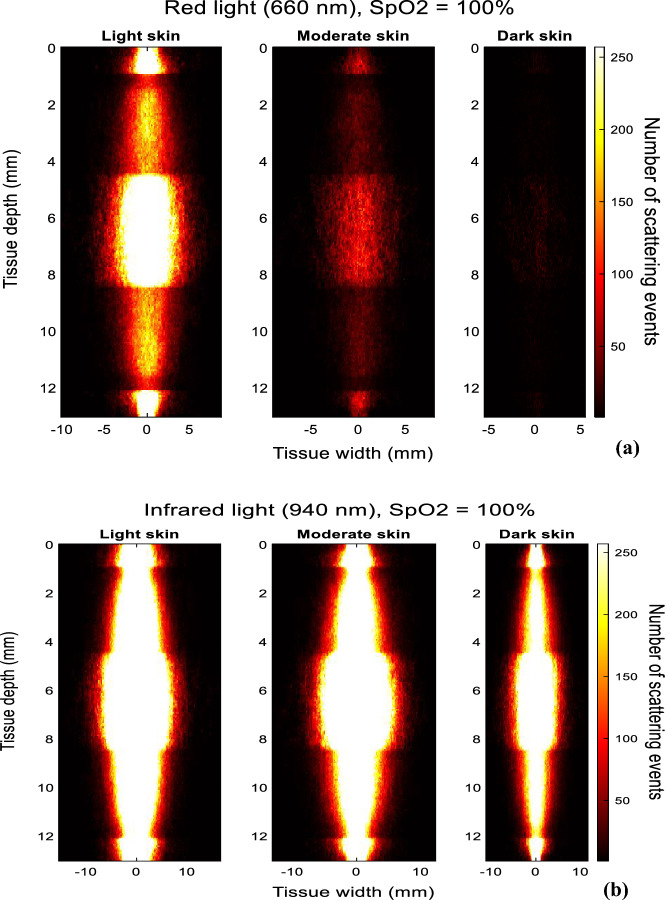
Photon propagation profiles of detected photons in transmittance PPG for light, moderate, and dark skin using a (**a**) 660 nm light source and (**b**) 940 nm light source.

The photon profiles of red and infrared light for light, moderate, and dark skin in transmittance PPG is presented in Fig. 4. By tracking and recording the trajectory of detected photons, the profiles were constructed using a histogram of 200 bins (n), which enabled direct comparisons in the behaviour of light in the finger model between the two wavelengths across the different skin types. From the data, out of the ten million red photons launched at the tissue surface, 1791 were detected by light skin, 341 by moderate skin, and 55 by dark skin. Conversely, in the case of infrared light, light, moderate, and dark skin detected 9655, 6846, and 4408 respectively, all during diastole. Notably, with red light, the number of detected photons varied significantly with melanin concentration, resulting in contrasting scattering patterns relative to infrared light. The scattering events were observed to be most pronounced in the dermal layers near the source and the photodetector, as well as the muscle and bone layer. The influence of melanin concentration on the number of detected photons and scattering events appeared to be less prominent with infrared light, suggesting the relatively minimal impact of skin type on photon propagation at longer wavelengths.

**Figure 5:**
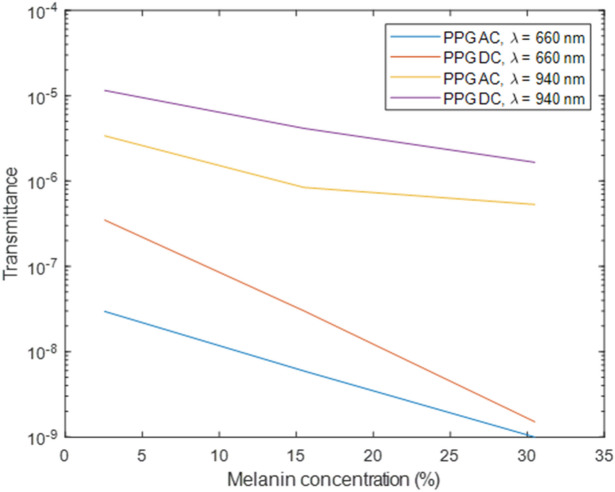
Calculated AC and DC transmittance values for light, moderate, and dark skin at 660 nm and 940 nm in logarithmic scale.

In Fig. 5, the results demonstrate the magnitudes of AC (alternating current) and DC (direct current) components for a static point in a PPG signal for red and infrared light across light, moderate, and dark skin. Transmittance, calculated by taking the ratio of detected intensity to the incident intensity, indicates the portion of light that passes through the finger model and is received by the photodetector. The data clearly illustrates that both AC and DC transmittance values decrease with increasing melanin concentration. As anticipated, the AC component is significantly smaller than the DC component at both wavelengths, reflecting the relatively small amplitude of AC outputs in PPG signals. However, the AC and DC values for red light are notably smaller compared to infrared light, and hence, a logarithmic scale is used to visualise the differences in transmittance as melanin concentration changes. The decreasing difference between the AC and DC components becomes evident as melanin concentration increases, and that overall, red light is most absorbed by dark skin (${v}_{mel}$ = 30.5%). Nevertheless, the AC and DC components exhibit a consistent difference in magnitude with increasing melanin concentration for infrared light, further suggesting its limited influence at this specific wavelength if 940 nm.

**Figure 6:**
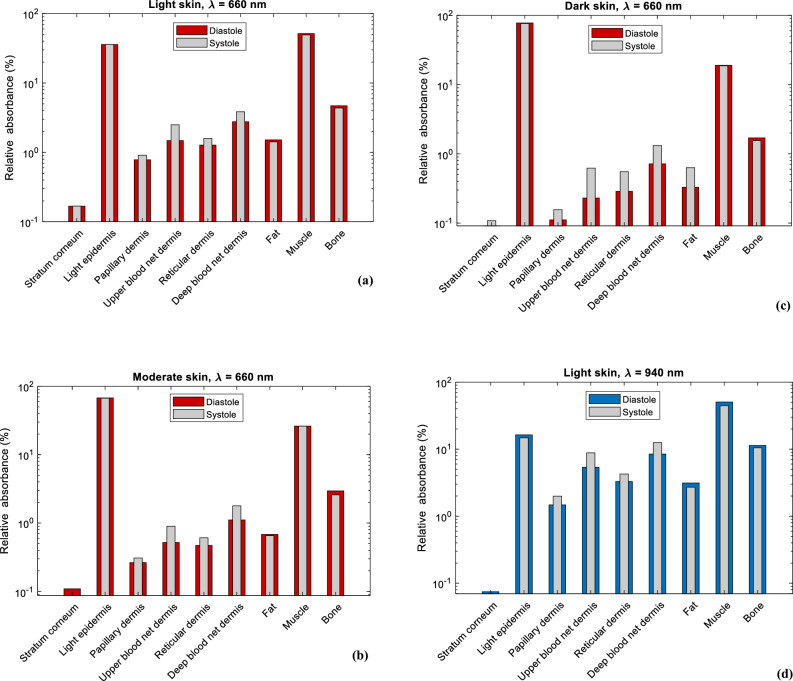

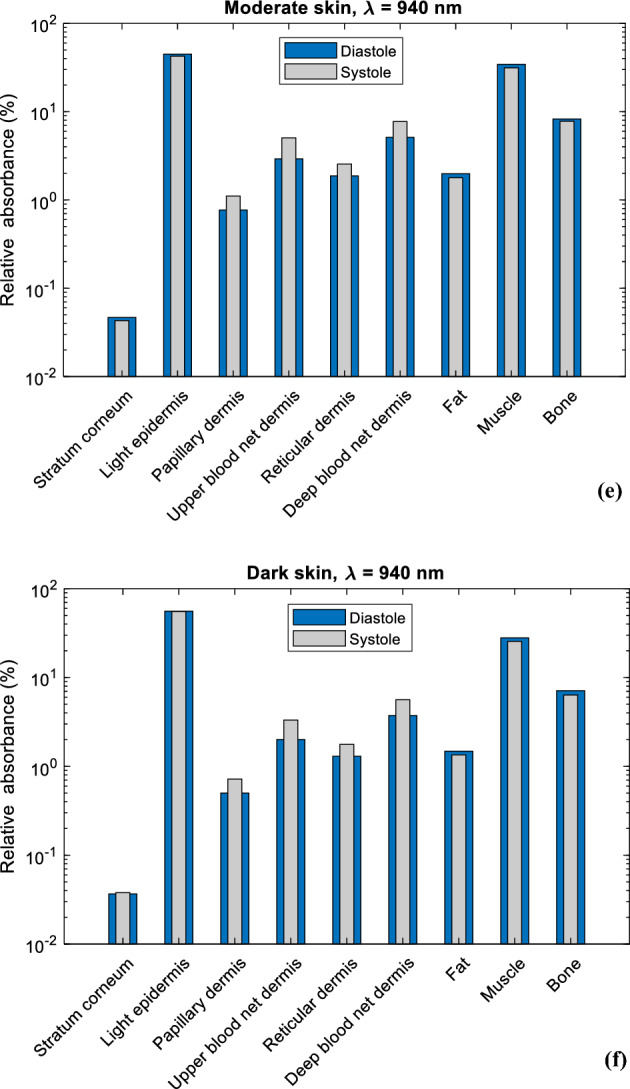
Relative absorbance of detected photons in each sublayer of the simulated finger model at diastolic and systolic states for light, moderate, and dark skin for (**a**–**c**) red light and (**d**–**f**) infrared light.

Moreover, the relative absorbance of detected photons per layer for red and infrared light in both systolic and diastolic states are presented in Fig. 6. Hence, the distribution of absorption in all the layers of the finger model are presented based on the concentration of melanin. For the epidermal layer, it is evident that the dark epidermis exhibits the highest relative absorbance, followed by the moderate epidermis and the light epidermis for both wavelengths. For red light, relative absorbance was approximately 78%, 68%, and 36% respectively, while for infrared light, relative absorbance was 56%, 45%, and 16% respectively, both during diastole. For all skin types, the stratum corneum is seen to have the lowest relative absorbance at both wavelengths. In the dermal layers, relative absorbance during systole is greater than diastole due to increased blood volume, which increases the capacity of the dermis to absorb more photons. Overall, as the relative absorbance in the epidermis increases due to increasing melanin concentration, the relative absorbance in the dermal layers as well as fat, muscle, and bone layers decreases.

### Reflectance PPG

**Figure 7:**
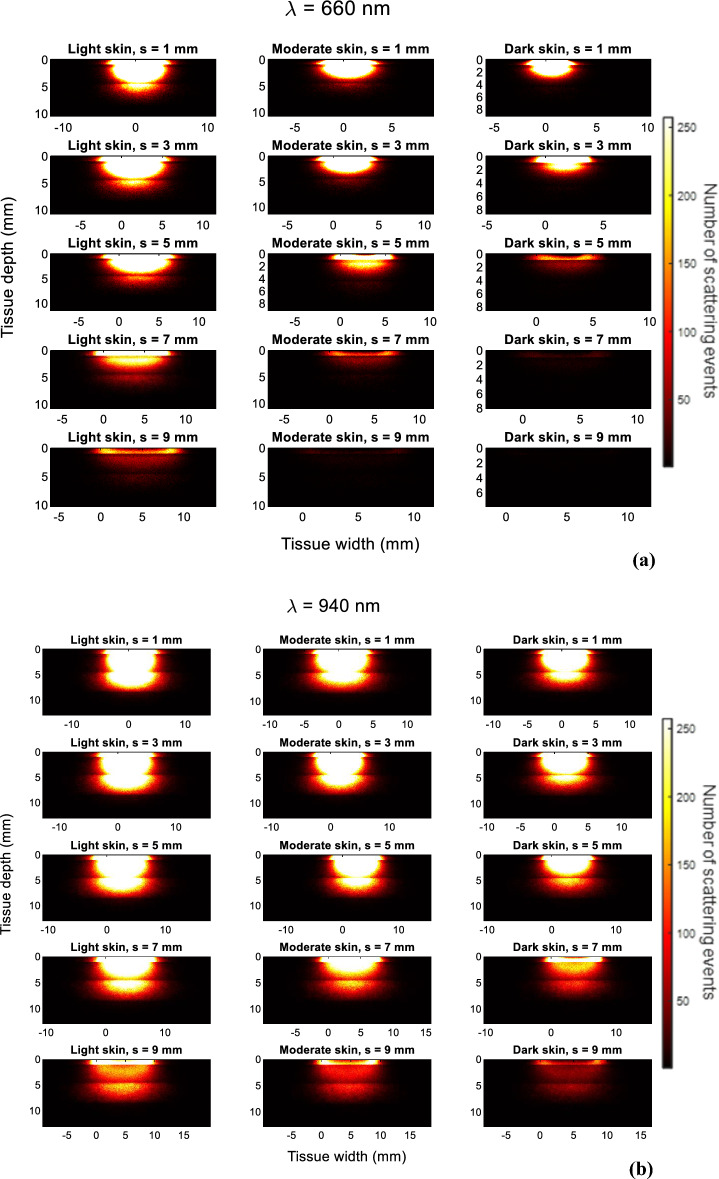
Photon propagation profiles of detected photons in reflectance PPG for light, moderate, and dark skin using a (**a**) 660 nm light source and (**b**) 940 nm light source across a source-detector separation range of 1–9 mm. Brighter regions illustrate the high density of scattering events.

In Fig. 7a and b, the photon profiles for light, moderate, and dark skin are presented for red and infrared light, respectively, across five different source-detector separations (1 mm, 3 mm, 5 mm, 7 mm and 9 mm). As the case with the photon profiles in transmission mode, only the trajectory of the photons that have been detected are shown, and not the photons that have been absorbed or internally reflected when they entered the tissue. The horizontal lines indicate the boundary of each layer of the finger model.

There is an inverse relationship between the source-detector separation and the number of detected photons and scattering events for each skin type. Additionally, photon density is immensely reduced starting from moderate skin when s = 5 mm and beyond. This implies that the photons are unable to travel long distances between the source and detector before being completely absorbed, a phenomenon that intensifies with higher concentrations of melanin.

For infrared light, slight differences in the scattering density are observed among the three skin types at smaller source-detector separations, such as 1 mm and 3 mm. Even when the separation is 9 mm, 4790 infrared photons are detected by dark skin in comparison to 173 red photons, indicating the greater probability of red-light absorption at higher melanin concentrations. In comparison to absorption, scattering is the more dominant mechanism of the light-tissue interactions with infrared compared to red light, irrespective of the skin type, even at large-source detector separations.

**Figure 8:**
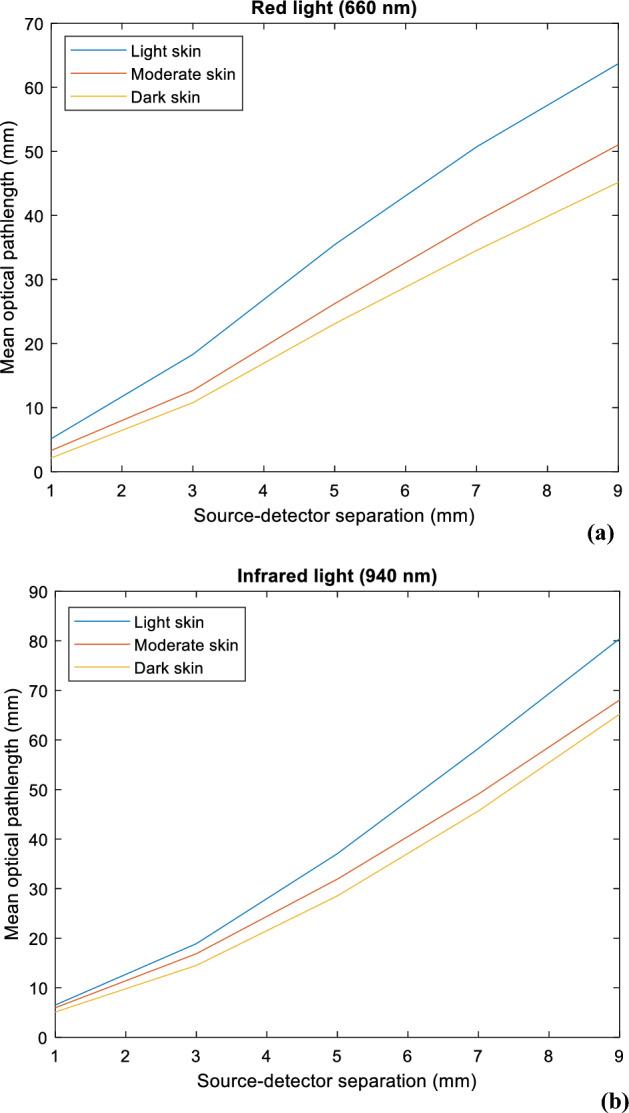
Mean optical pathlength for light, moderate, and dark skin across a source-detector separation range of 1–9 mm at (**a**) 660 nm and (**b**) 940 nm.

**Figure 9:**
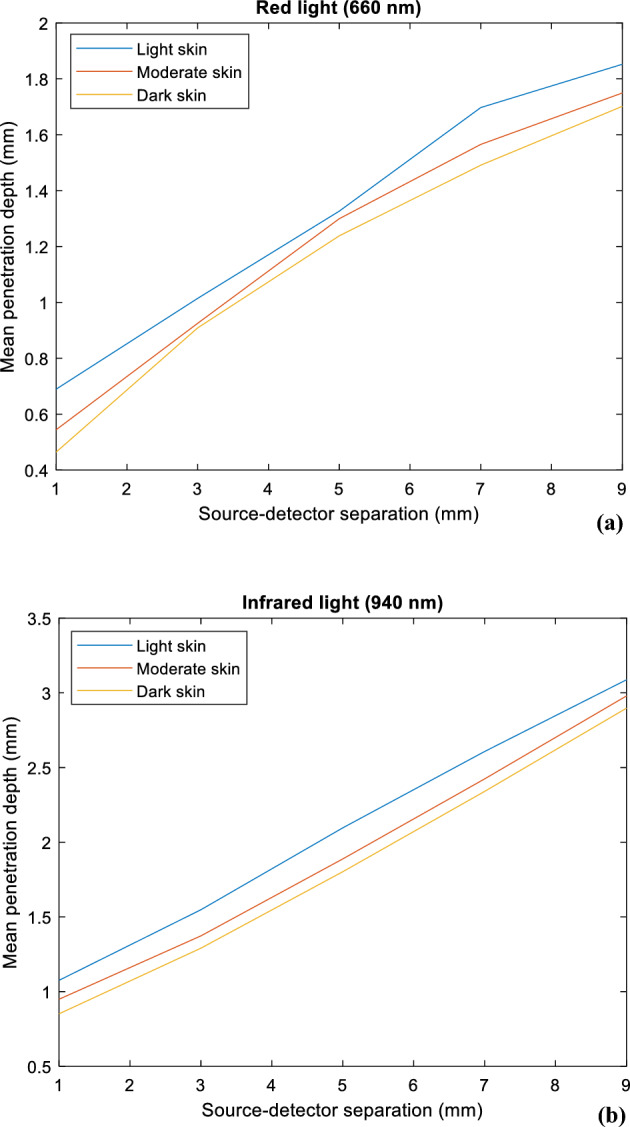
Mean photon penetration depth for light, moderate, and dark skin across a source-detector separation range of 1–9 mm at (**a**) 660 nm and (**b**) 940 nm.

Figure 8 provides information on the mean optical pathlength for each skin type at both red and infrared wavelengths, against a range of source-detector separations between 1 and 9 mm. At both wavelengths, there is a directly proportional relationship between the source-detector separation and the mean optical pathlength, and an inversely proportional relationship between melanin concentration and mean optical pathlength. However, it is important to note that the mean optical pathlength is consistently higher for infrared light compared to red light, as indicated by the respective magnitude ranges on the y-axes of the figures. This suggests that, on average, there is a greater tendency for infrared photons to scatter, which increases the total distance they travel before reaching the photodetector, regardless of skin type. These findings correlate directly with the behaviour of the photons observed in Fig. 9, showing the increase in photon penetration depth as the source-detector separation increases for both red and infrared wavelengths. As expected, the range of penetration depth is greater for infrared light than red light, as photons have travelled a longer distance.

**Figure 10:**
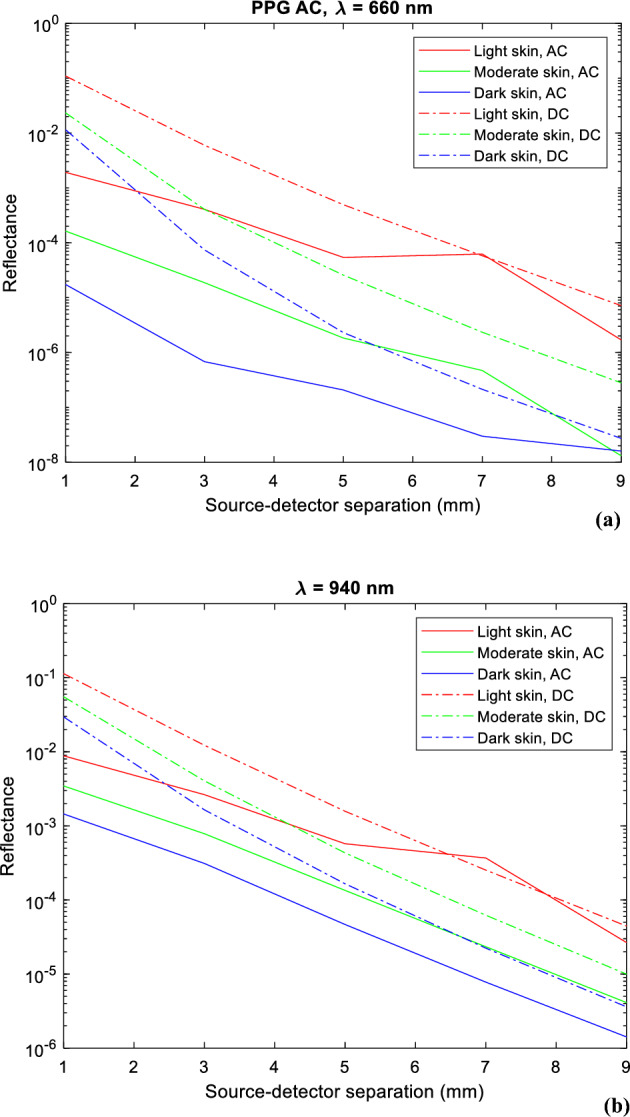
Calculated AC and DC reflectance values for light, moderate, and dark skin across a source-detector separation range of 1–9 mm. (**a**) AC and DC, 660 nm and (**b**) AC and DC, 940 nm.

Lastly, Fig. 10 presents the AC and DC components of an instantaneous point in a simulated PPG signal for red and infrared light. Results for light, moderate, and dark skin are shown as the source-detector separation varies from 1 to 9 mm. Similar to the previous observations with transmittance AC and DC (Fig. 3), the results reveal how the reflectance AC and DC components of the PPG are compromised as a result of increased melanin concentration. Notably, there is a sharp decline in the DC component at both wavelengths beyond a source-detector separation of 1 mm, and an even significant drop in the AC component beyond 5 mm. However, the difference between these PPG components in transmittance and reflectance lies in their magnitudes, ranging between ${10}^{-5}$ and ${10}^{-7}$ for transmittance and ${10}^{-2}$ and ${10}^{-3}$ for reflectance.

### Statistical analysis

The following section presents an evaluation of the results presented of in Sects. “Transmittance PPG” and "Reflectance ppg” for further analysis. The specific outputs of interests are (a) the number of detected photons in transmittance and reflectance PPG, (b) mean optical pathlength in reflectance PPG, (c) mean penetration depth in reflectance PPG and, (d) AC/DC ratio in reflectance mode.

To evaluate the certainty of the simulations, the convergence rate (Q), given by the inverse square root of the number of detected photons (N), is calculated to select the results to be further discussed and analysed. Therefore, any results with a convergence rate less than 0.001 (N $<$ 1,000,000), were omitted, including all those in transmittance (Table 4). Calculation of the AC/DC ratio was completed for all skin types in reflectance when s = 1 mm and 3 mm.

**Table 4:** The number of detected photons and convergence rate in transmittance mode for light, moderate, and dark skin at 660 nm and 940 nm.

Transmittance PPG						
	Number of detected photons (percentage as compared to light skin at the respective wavelength, %)			Convergence rate (Q)		
	Light	Moderate	Dark	Light	Moderate	Dark
660 nm	1791 (100)	341 (19)	55 (3.1)	0.024	0.054	0.135
940 nm	9655 (100)	6846 (71)	4408 (46)	0.010	0.012	0.015

For the analysis of transmittance PPG, the number of detected is indicative of the amplitude and quality of the PPG signal. A reduction in the number of detected photons is observed for both red and infrared light across the three skin types. However, this decrease is more pronounced at 660 nm, evident by the smaller percentage values in moderate and dark skin pigmentation relative to light pigmentation (19% and 3.1% vs. 71% and 46%). This aligns with the decline in the DC intensity, resulting from increased levels of absorption of greater concentrations of melanin.

**Figure 11:**
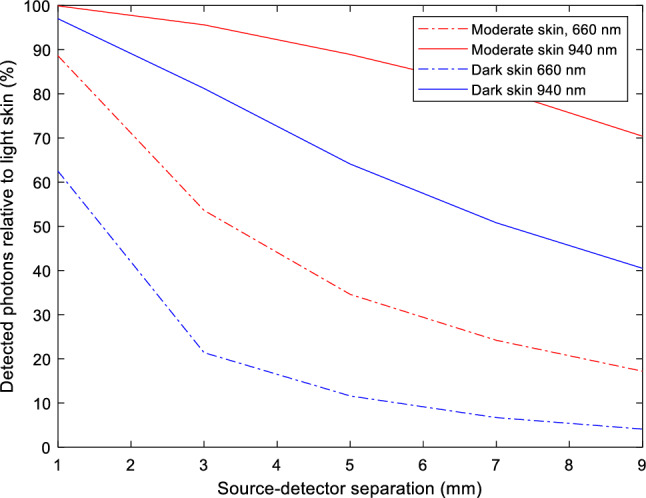
Number of photons detected by moderate and dark skin relative to light skin at 660 nm and 940 nm (%).

Furthermore, similar trends in the number of detected photons are observed in reflectance mode across the selected source-detector distances (Fig. 11). Firstly, the relative percentage range of detected photons in moderate and dark skin is notably greater in comparison to transmittance PPG. This results from photons being less susceptible to repeated absorption and scattering due to the relatively short distances in which they have travelled (in comparison to 13 mm in transmission).

Moreover, the statistics also present the decline in detected photon count for moderate and dark skin, relative to light skin, at both wavelengths, as the source-detector separation increases. This demonstrates the influence of the separation distance and changes in melanin concentration on the accuracy of the Monte Carlo model. In the context of finger probe designs, which have been seen to produce high-quality PPG signals at separation distances between 2 and 6 mm^39^, the relative percentage decrease in detected photons becomes a critical factor, particularly for dark skin at 660 nm.

Determining the percentage difference between two data sets provides insight into their proximity (inferred by a small percentage difference), or divergence (inferred by a large percentage difference). Tables 5 and 6 display the relative percentage differences in the mean penetration depth and mean optical pathlength among the three skin type combinations, wavelengths, and source-detector separations.

**Table 5:** Percentage difference in mean penetration depth between light, moderate, and dark skin across a source-detector separation range of 1–9 mm (%).

Wavelength (nm)	Source-detector separation (sds, mm)	Skin type combination	Percentage difference in mean penetration depth (%)					Mean % difference across all sds
			1	3	5	7	9	
660		Light–Moderate	5.89	2.30	0.516	2.02	1.43	2.43
		Light–Dark	9.76	2.75	1.72	3.23	2.12	3.92
		Moderate–Dark	3.96	0.447	1.21	1.22	0.692	1.51
940		Light–Moderate	3.13	2.99	2.61	1.82	0.892	2.29
		Light–Dark	5.82	4.53	3.76	2.69	1.59	3.68
		Moderate–Dark	2.71	1.55	1.16	0.877	0.697	1.39

**Table 6:** Percentage difference in mean optical pathlength between light, moderate, and dark skin across a source-detector separation range of 1–9 mm (%).

Wavelength (nm)	Source-detector separation (sds, mm)	Skin type combination	Percentage difference in mean optical pathlength (%)					Mean % difference across all sds
			1	3	5	7	9	
660		Light–Moderate	10.8	9.03	7.57	6.47	5.52	7.88
		Light–Dark	20.5	12.9	10.6	9.48	8.51	12.4
		Moderate–Dark	10.7	4.07	3.18	3.09	3.05	4.81
940		Light–Moderate	2.26	2.84	3.72	4.28	4.16	3.45
		Light–Dark	6.09	6.58	6.49	6.07	5.22	6.09
		Moderate–Dark	3.86	3.77	2.79	1.81	1.07	2.49

When taking measurements from a finger using a PPG reflectance sensor, a source-detector separation of 3 mm is considered as an optimal distance to minimise saturated light^39^. At this distance, there is a 2.75% difference in mean penetration depth between light and dark skin for red light, and 4.53% for infrared light. This indicates that penetration depth is limited irrespective of melanin concentration, relative to the greater depth in which infrared light can travel in light skin. Overall, moderate-dark skin is seen to exhibit the lowest percentage, while the highest difference is observed between light–dark skin at both wavelengths. This aligns with the approximately 2 and 12 times increase in melanin concentration between the two skin group combinations, respectively.

Furthermore, the percentage difference in mean optical pathlength between light and dark skin is almost 2.5 times greater than that between moderate and dark skin at both wavelengths. Evidently, dark skin yields the shortest optical pathlength as a result of its higher absorption rate, causing photons to be terminated more rapidly compared to light and moderate skin. However, this relationship gradually weakens with an increase in the source-detector separation, and suggests that there is no one correlation between photon pathlength and penetration depth for all wavelengths.

**Figure 12:**
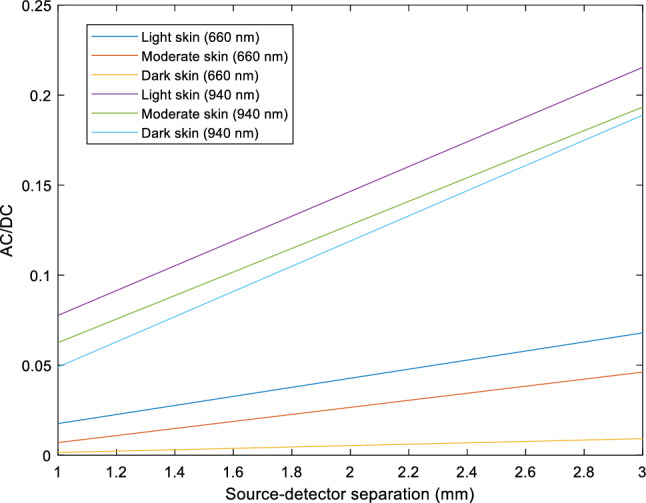
AC/DC ratio for light, moderate, and dark skin at source-detector separations of 1 mm and 3 mm for red (660 nm) and infrared (940 nm) light.

Lastly, the AC/DC ratios across the three skin types, both wavelengths, and at source-detector separations of 1 mm and 3 mm, are shown in Fig. 12. For red and infrared light, the AC/DC ratio is highest for light skin, followed by moderate skin, and then dark skin. Notably, the AC/DC ratios are higher at 940 nm compared to 660 nm, with a narrower difference in magnitude between the three skin types. It becomes evident from Fig. 10 that skin type, particularly with red light, significantly reduces AC reflectance in comparison to infrared light, resulting in a larger gap between the AC and DC components. Consequently, when a smaller AC value is divided by a relatively similar (and larger) DC value between all skin types, it results in an overall reduced ratio. This suggests that the AC has a greater impact on the AC/DC ratio resulting from changes in melanin concentration. Since the AC/DC ratio is an important parameter in photoplethysmography that is often analysed to assess the strength or amplitude of the pulsatile component, changes in this ratio due to differences in skin pigmentation must be accounted for. Therefore, while a reduced AC/DC ratio may indicate poor pulsatile signal quality, potentially due to weakened arterial flow, in this instance, it may be due to interference from the patient’s skin colour.

## Discussion

This paper presented a robust Monte Carlo simulation to evaluate the impact of melanin concentration on PPG signals. In the field of biomedical optics, in-silico modelling has proven to be crucial for understanding how light interacts with tissue and the various factors that contribute to the PPG waveform, which still contains many unknowns. However, computational models often overlook uncontrollable factors in experimental setups such as movement artifacts, and can only replicate certain factors to a relative degree. Pigmentation is a complex component of skin, influenced by multiple intrinsic and extrinsic factors^40^. It is determined by the quantity and distribution of various skin chromophores, including melanin, keratin, carotene, haemoglobin, and water, which vary between individuals, even if they appear to have the same skin pigmentation to the human eye. Consequently, to accurately implement skin pigmentation through a computational model can be challenging, especially due to the limited research conducted on variability in skin properties on large groups of people and/or with similar sample sizes in each pigmentation/ethnic group. To address this challenge, the most practical approach was to employ the Fitzpatrick scale, the most widely used and FDA-approved method for stratifying skin pigmentation. This was achieved by calculating the absorption coefficient of the epidermal layer by changing the concentration of melanin, the primary determinant of skin pigmentation. Although dark skin has an absorption coefficient that is two and eleven times more than light and moderate skin respectively at both wavelengths (Table 3), the differences in the transmittance photon profiles, including scattering density and detection count between the two wavelengths, are visually apparent.

Furthermore, the accuracy of the model can be assessed by calculating its convergence rate, determined by the inverse of the square root of the number of simulated photons (Q). Simulating ${10}^{7}$ photons was a sufficient amount to yield reliable and consistent results that adhered to the principles of tissue optics at shorter source-detector separate (1 mm and 3 mm). Additionally, the model was optimised to launch photons simultaneously, leveraging the computational resources available in order to enhance simulation speed.

The human finger was chosen as the anatomical region of interest to model in the MC simulation due to its practicality and popularity in assessing peripheral perfusion, and the common site for pulse oximetry, an application based on the principles of photoplethysmography. Hence, this led to focus on investigating the light-tissue interactions at the two operating wavelengths used in pulse oximetry: red and infrared light. The growing attention given to the impact of skin pigmentation on the accuracy of pulse oximeters, especially during and after the COVID-19 pandemic, has sparked curiosity and motivation to understand the potential factors that contribute to the variation in PPG measurements based on skin pigmentation. Although the qualitative and quantitative data has highlighted substantial disparities between light, moderate and dark skin, it is important to note that pulse oximetry is concerned with the ratio of the outputs between red and infrared light, hence, the data must be evaluated depending on the application. Also, whilst most finger probes rely on transmittance PPG, there has been a rise in the use of health wearables among consumers, which employ predominantly reflectance PPG. Therefore, it was essential to study the effect of melanin concentration in both sensor configurations.

It was observed that melanin, particularly at higher concentrations, absorbed more red light than infrared light in both transmittance and reflectance PPG modes. As a consequence, the magnitudes of the AC and DC components in the red-light PPG signals were smaller compared to the infrared light signals. The characteristics of the epidermis, especially the absorption coefficient, significantly influence how red and infrared light are absorbed. The initial step size that a photons travel is calculated with a random number ($\xi$) between 0 and 1. As the photons are assumed to be orthogonally incident on the finger model, this random number must equal to or be less than 0.5985 and 0.7306 for red and infrared light respectively, to surpass the stratum corneum and reach the epidermis. Consequently, there is a greater probability for infrared light to initially localise in the epidermis. However, despite this, the optical characteristics of the epidermis at 660 nm, particularly with increased melanin concentration, have a more pronounced influence on the behaviour of light in tissue and subsequently impact the generated bio-optical outcomes.

Moreover, the data indicated that increasing the source-detector separation led to a reduction in the intensity of both PPG components. The differences in magnitude between transmittance and reflectance AC and DC imply distinct measurement characteristics and sensitives to changes in skin chromophores, including melanin. However, it is important to note that transmittance PPGs are generally more accurate than reflectance PPGs since they exhibit a higher probability of measuring absorption in all sublayers of the anatomical site.

In principle, there is no reason for the AC/DC ratio to be affected by melanin absorption because the contribution of melanin absorption to systolic absorbance is cancelled out with the contribution of melanin absorption to diastolic absorbance under Beer Lambert’s law. However, it may be, that there is a threshold to which light can behave similarly or within a narrow deviation across all skin types and in accordance with optical theory. This is similar to the significant contrast observed in absorption and scattering behaviours between red and infrared light, despite dark skin having an absorption coefficient 11 times higher than light skin at both wavelengths. Hence, information that is derived from PPG measurements must account for differences in skin pigmentations, especially those that are taken using smaller wavelengths.

The photon profiles revealed the significant prevalence of scattering when simulating infrared light, despite dark skin having an 11-fold higher photon absorption capacity per mm compared to light skin. In reflectance mode, only increases in the source-detector separation appeared to decrease the number of scattering events, enabling photons to penetrate deeper into the finger model and thus increasing their optical pathlength. These findings underscore the importance of considering the source-detector separation when studying depth of photon penetration. Although the relationship between penetration depth, optical pathlength, source-detector spacing and near infrared spectroscopy (NIRS) is known^41^, this study has shown additionally the effect of melanin concentration on these optical parameters. Consequently, they can be accounted for in the design and optimisation of sensors to minimise the influence of skin pigmentation in PPG measurement, which may enhance the accuracy and reliability of diagnostics, health monitoring, and treatment.

The current model has provided valuable insights into the interaction between red and infrared light and varying melanin concentrations, allowing for the preliminary replication of different skin pigmentations. The study’s exploration of light-tissue interactions provides significant benefits by explaining the extent of light absorption in transmittance versus reflectance across three skin types. This understanding holds implications for computational resources in simulations aimed at analysing skin pigmentation's impact on PPG -based applications, such the issue of overestimated arterial oxygen saturation (${{\text{SpO}}}_{2}$) in individuals with darker skin pigmentation as reported in the recent literature^42–44^. For example, in future simulations, an equal and substantial number of photons need to be detected in both transmittance and reflectance PPG modes across all skin types. This ensures the development of a more robust model, addressing the challenge of skin pigmentation in biosensor devices before delving further into its possible complexities.

As previously stated, a number of clinical and computational studies have focused on quantifying the combined effects of melanin concentration and oxygen saturation to understand, conclusively, the effect of skin pigmentation on the accuracy of pulse oximeters^6,21^. However, the motivation of this study is to explore individually the behaviour of confounding factors influencing photoplethysmography signals and to assess their relative impact. With this, the study delves into the fundamental science underpinning the ongoing concern of the impact of skin pigmentation on the accuracy of physiological measurements acquired from a range of biosensor technologies. Hence, while this study has the potential to provide more clinically relevant application-based data, such as the calculation of the ratios of ratios in the context of pulse oximetry, the current findings offer valuable insights into the behaviour of light in three distinct skin colours from ‘healthy’ individuals. These results can then serve as a reference for future simulations when additional confounding factors are incorporated into the model.

## Conclusions

Photoplethysmography has revolutionised health monitoring, however some of its limitations, specifically skin pigmentation, has not yet been fully addressed. This study has demonstrated the significance of melanin concentration in influencing the interactions between light and tissue in PPG measurements. Developing an in-silico Monte Carlo model of the human finger and changing the melanin concentration in an attempt to replicate different skin pigmentation has highlighted the effect on the PPG signal components and other parameters. These insights shed light on the impact of melanin on PPG performance and provide the foundation for addressing the disparities observed in PPG measurements among individuals with different skin pigmentation, ultimately enhancing the accuracy and reliability of PPG-based healthcare devices.

## Data Availability

The datasets generated during and/or analysed during the current study are available from the corresponding author on reasonable request.
